# Etiology-associated heterogeneity in acute respiratory distress syndrome: a retrospective cohort study

**DOI:** 10.1186/s12890-021-01557-9

**Published:** 2021-05-31

**Authors:** Sheng-Yuan Ruan, Chun-Ta Huang, Ying-Chun Chien, Chun-Kai Huang, Jung-Yien Chien, Lu-Cheng Kuo, Ping-Hung Kuo, Shih-Chi Ku, Huey-Dong Wu

**Affiliations:** Division of Pulmonary and Critical Care Medicine, Department of Internal Medicine, National Taiwan University Hospital and College of Medicine, No. 7, Chung-Shan South Road, Taipei, 10002 Taiwan

**Keywords:** Acute respiratory distress syndrome, Heterogeneity, Phenotype, Pneumonia, Respiratory failure, Sepsis

## Abstract

**Background:**

Heterogeneity in acute respiratory distress syndrome (ARDS) has led to many statistically negative clinical trials. Etiology is considered an important source of pathogenesis heterogeneity in ARDS but previous studies have usually adopted a dichotomous classification, such as pulmonary versus extrapulmonary ARDS, to evaluate it. Etiology-associated heterogeneity in ARDS remains poorly described.

**Methods:**

In this retrospective cohort study, we described etiology-associated heterogeneity in gas exchange abnormality (PaO_2_/FiO_2_ [P/F] and ventilatory ratios), hemodynamic instability, non-pulmonary organ dysfunction as measured by the Sequential Organ Failure Assessment (SOFA) score, biomarkers of inflammation and coagulation, and 30-day mortality. Linear regression was used to model the trajectory of P/F ratios over time. Wilcoxon rank-sum tests, Kruskal–Wallis rank tests and Chi-squared tests were used to compare between-etiology differences.

**Results:**

From 1725 mechanically ventilated patients in the ICU, we identified 258 (15%) with ARDS. Pneumonia (48.4%) and non-pulmonary sepsis (11.6%) were the two leading causes of ARDS. Compared with pneumonia associated ARDS, extra-pulmonary sepsis associated ARDS had a greater P/F ratio recovery rate (difference = 13 mmHg/day, *p* = 0.01), more shock (48% versus 73%, *p* = 0.01), higher non-pulmonary SOFA scores (6 versus 9 points, *p* < 0.001), higher d-dimer levels (4.2 versus 9.7 mg/L, *p* = 0.02) and higher mortality (43% versus 67%, *p* = 0.02). In pneumonia associated ARDS, there was significant difference in proportion of shock (*p* = 0.005) between bacterial and non-bacterial pneumonia.

**Conclusion:**

This study showed that there was remarkable etiology-associated heterogeneity in ARDS. Heterogeneity was also observed within pneumonia associated ARDS when bacterial pneumonia was compared with other non-bacterial pneumonia. Future studies on ARDS should consider reporting etiology-specific data and exploring possible effect modification associated with etiology.

**Supplementary Information:**

The online version contains supplementary material available at 10.1186/s12890-021-01557-9.

## Background

Acute respiratory distress syndrome (ARDS) is a clinical syndrome of inflammatory lung injury characterized by non-cardiogenic lung edema, severe hypoxemia and impaired lung mechanics [1, 2]. Clinicians and researchers use a valid operational definition to identify patients with pathophysiological features of ARDS and implement clinical practice guidelines [2]. A wide variety of etiologies, referred to as precipitating risk factors in the literature, can lead to ARDS [2, 3]. Pneumonia is the most common etiology of ARDS and accounts for roughly half of all ARDS cases [4, 5]. Other common etiologies include extrapulmonary sepsis, aspiration, noncardiogenic shock, transfusion and trauma [4, 5]. Different etiologies of ARDS can result in different histological and biological changes in the lungs [6, 7].

Cumulative data have suggested that ARDS is a heterogeneous syndrome with diverse radiographic lung morphology, respiratory mechanics and biomarker profiles [8, 9]. The heterogeneity of ARDS may explain the negative results observed in many clinical trials [10–12]. To combat this heterogeneity, researchers and clinicians have been working on phenotyping to help identify homogenous subsets of ARDS [13, 14]. Understanding the source of heterogeneity is a crucial step in phenotyping. The etiology of ARDS is considered an important source of heterogeneity [15, 16]; however, previous studies have usually adopted a dichotomous classification to evaluate etiology-associated heterogeneity, such as pulmonary versus extrapulmonary ARDS or sepsis versus non-sepsis ARDS [17, 18]. Data for direct comparisons between individual etiologies for clinically important variables, such as gas exchange indexes, hemodynamic stability and biomarkers, remains limited. Whether there are between-etiology differences in these variables may have implications for ARDS management because these factors are potential effect modifiers for high positive end-expiratory pressure (PEEP), recruitment maneuvers, prone positioning and pharmacological interventions, such as steroids [8, 19–21].

We hypothesized that etiology is an important source of heterogeneity in ARDS and partly accounts for the diversity of clinical course, organ damage and outcomes in patients with ARDS. This study aimed to explore the etiology-associated heterogeneity in ARDS by examining the differences between major etiologies of ARDS in terms of gas exchange, hemodynamics, non-pulmonary organ dysfunction, biomarkers, and mortality. We also evaluated the differences between bacterial and non-bacterial pneumonia associated ARDS because pneumonia accounts for half of all ARDS cases [4].

## Methods

### Study design and data source

This retrospective cohort study was conducted at the National Taiwan University Hospital in Taiwan, and aimed to explore the potential heterogeneity associated with ARDS etiologies by comparing and evaluating gas exchange abnormality, hemodynamic instability, non-pulmonary organ dysfunction, inflammation and coagulation biomarkers, and mortality. The Strengthening the Reporting of Observational Studies in Epidemiology (STROBE) guidelines were followed. We used a patient list from a quality improvement program to early identify mechanically ventilated patients with a PaO_2_/FiO_2_ (P/F) ratio ≤ 300 mm Hg in the ICU. The medical records and chest radiographs for these patients were reviewed to obtain the data required for this study.

In September 2014, a quality improvement program was initiated in the study hospital to enable early recognition of acute lung injury in eight ICUs. Respiratory therapists actively screened ventilated patients to see whether their P/F ratios had been ≤ 300 mm Hg for > 12 h. Once a patient fulfilled these criterion, the in-charge doctor was notified by email. The doctor was then invited to voluntarily answer a web-based questionnaire regarding whether the case fulfilled the four domains of the Berlin definition for ARDS [2]. The procedure ended after the e-mail notification and further management was at the discretion of the primary care doctors.

### Establishment of the ARDS cohort

Using the aforementioned P/F ratio ≤ 300 mm Hg data, we identified cases of initiating invasive mechanical ventilation between October 2014 and November 2015 for analysis. Two pulmonologists independently reviewed the medical records and chest radiographs of these patients to evaluate whether they fulfilled the timing, chest imaging, origin of edema and oxygenation criteria for ARDS, according to the Berlin definition [2]. The etiology of hypoxemia and a diagnosis of ARDS were determined by a discussion between reviewers. Patients were followed up from the first day that their P/F ratio was ≤ 300 mm Hg until death or hospital discharge whichever occurred first.

### Data collection

To determine and describe heterogeneity in ARDS, we collected data on (1) gas exchange, (2) shock and non-pulmonary organ dysfunction, (3) inflammation and coagulation biomarkers, and (4) 30-day mortality. These variables were selected based on the available data and their relevance to patient management in ARDS. Specifically, we collected data on arterial blood gas and ventilator settings (ventilator mode, FiO_2_, mean airway pressure, PEEP, and minute ventilation) in the morning of the first seven days to calculate P/F ratios and ventilatory ratios. Respiratory resistance and compliance, and individual organ system scores for the Sequential Organ Failure Assessment (SOFA) were collected on days 1, 3, 5 and 7. Baseline C-reactive protein (CRP), lactate dehydrogenase (LDH), albumin, d-dimer and lactic acid levels, comorbidities, and vital status at ICU and hospital discharge were also collected. Ventilatory ratio was defined as [minute ventilation (ml/min) × PaCO_2_ (mmHg)]/(predicted body weight × 100 × 37.5) [22]. We also collected microbiology testing data. Detailed microbiological data is provided in Additional file 1: Table S1.

### Missing data and imputation

Inherent to the nature of the retrospective study design, there was a varied range of missing data for the collected variables. The proportion of missing data is summarized in Additional file 1: Tables S1 and Table S2. Because missing data may affect the representativeness of our results, imputation was performed for the missing P/F ratios and SOFA scores. We used the last-observation-carried-forward method to replace the missing data with substituted values when the missing data occurred on day 2 onwards. If the missing data occurred on day 1 for any one of the six organ system SOFA scores, a zero point was assigned to that organ system score. The rationale behind this imputation strategy was that intensivists tend not to order tests to evaluate organ systems when they appear clinically normal.

### Statistical analysis

Data were presented as the number with proportion, mean with standard deviation (SD) or median with inter-quartile range (IQR) as appropriate. To describe heterogeneity in ARDS, we compared differences in gas exchange abnormality (P/F ratios and ventilator ratios), shock and non-pulmonary organ dysfunction, inflammation and coagulation biomarkers, and 30-day mortality between the major etiologies of ARDS. Chi-squared tests, Wilcoxon rank-sum tests and Kruskal–Wallis rank tests were used to compare the differences between ARDS etiologies. We used linear regression to model the trajectories of P/F ratios over time. We added an interaction term (etiology x time) to the regression model to test whether the P/F ratio trajectories were different between etiologies.

We used Stata software version 15 (StataCorp, College Station, TX, USA) for statistical analysis. Statistical tests were two-sided and a *p*-value of < 0.05 was considered to indicate a statistically significant difference. To estimate the sample size, we assumed that pneumonia and extra-pulmonary sepsis were two major causes of ARDS, accounting for 60% and 20% cases of ARDS, respectively [4]. Thus, a sample of 255 patients with ARDS would have 80% power to detect a 25% difference in 30-day mortality between pneumonia and extra-pulmonary sepsis associated ARDS at a two-sided type I error of 5%.

## Results

### Patient selection and characteristics

**Fig. 1:**
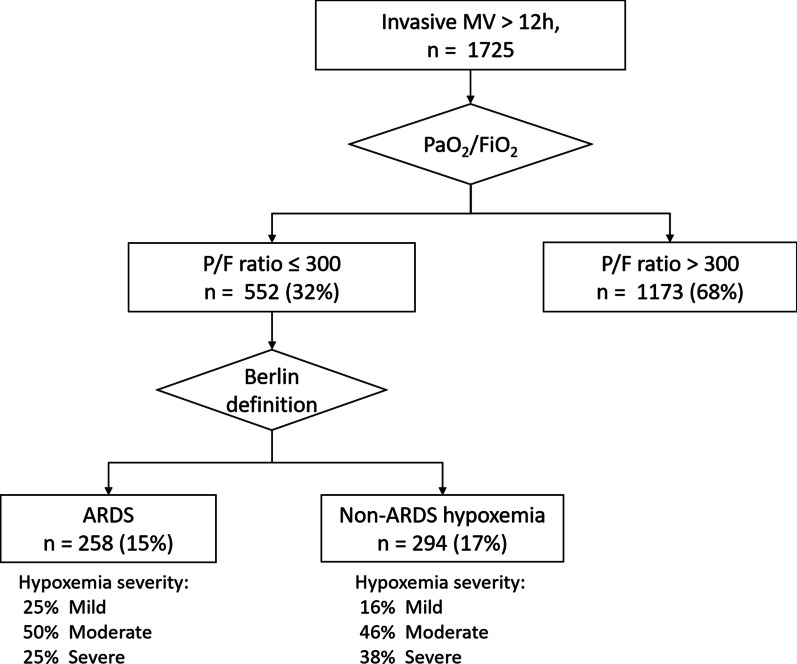
The selection process in this study and the case number at each stage

During the study period, there were 1725 patients who received invasive mechanical ventilation for > 12 h in the ICU (Fig. 1). Among them, 552 (32%) had severe hypoxemia with P/F ratios ≤ 300 mm Hg. Of these 552 patients with severe hypoxemia, 258 (47%) had ARDS and 294 (53%) had non-ARDS hypoxemia. Table 1 shows the baseline characteristics of the ARDS and non-ARDS cohorts. There were significant differences between the ARDS and non-ARDS groups in terms of their comorbidity profiles, gas exchange abnormalities and biomarkers. For the ARDS cohort, the median age was 67 years (IQR, 55–76), 68% were male and the P/F ratio on day 1 was 143 mm Hg (IQR, 99–200). The distribution of hypoxemia severity was 25% mild, 50% moderate and 25% severe (Fig. 1).

**Table 1:** Baseline characteristics of 552 patients with PaO_2_/FiO_2_ ratios ≤ 300 mm Hg

Characteristics	PaO_2_/FiO_2_ ≤ 300 mm Hg	
	ARDS (n = 258)	Non-ARDS (n = 294)
Age, year, median (IQR)	67 (55–76)	68 (59–80)
Sex, female, n (%)	83 (32)	106 (36)
Body mass index, median (IQR)	22.8 (20–26)	23.9 (20.7–27.6)
SAPS II score, median (IQR)	49 (40–57)	47 (39–57)
Comorbidities, n (%)		
Cancer	111 (43)	106 (36.1)
Cardiovascular diseases	58 (22.5)	110 (37.4)
Chronic obstructive airway diseases	22 (8.5)	34 (11.6)
Liver cirrhosis	20 (7.8)	19 (6.5)
Chronic kidney diseases	49 (19)	77 (26.2)
Diabetes	66 (25.6)	109 (37.1)
Autoimmune diseases	24 (9.3)	12 (4.1)
Respiratory parameters, median (IQR)		
FiO_2_	0.63 (0.5–1.0)	0.6 (0.45–0.8)
PaO_2_/FiO_2_ ratio, mm Hg	143 (99–200)	169 (121–226)
PEEP, cm H_2_O	8 (6–10)	6 (5–8)
pH	7.42 (7.37–7.45)	7.41 (7.36–7.45)
PaCO_2_, mm Hg	32 (28–37)	34 (29–41)
HCO_3_^−^, mmol/L	21 (18–24)	22 (19–25)
Tidal volume/pBW, mL/kg	8.6 (7.3–10.3)	8.2 (6.8–9.8)
Minute ventilation, L/min	10.5 (8.2–13.1)	8.3 (6.5–11.2)
Respiratory compliance, mL/cm H_2_O	30 (25–40)	30 (23–39)
Respiratory resistance, cm H_2_O‧s/L	15 (12–18)	17 (13–20)
Biomarkers, median (IQR)		
C-reactive protein, mg/dL	14.4 (8–20.4)	5.8 (1.8–12.8)
Platelet, K/μL	140 (72–208)	154 (91–224)
D-dimer, mg/L	4.5 (2.3–11.8)	7.0 (2.4–27.0)
Lactate dehydrogenase, U/L	428 (316–734)	368 (215–612)
Lactic acid, mmol/L	2.3 (1.5–4.2)	2.8 (1.5–5.5)
Albumin, g/dL	2.7 (2.3–2.9)	2.9 (2.5–3.3)

*IQR* interquartile range, *SAPS* simplified acute physiology score

### Etiologies of ARDS and non-ARDS hypoxemia

Table 2 summarizes the causes of ARDS and non-ARDS hypoxemia. Pneumonia was the leading cause of ARDS (48.4%), followed by extra-pulmonary sepsis (11.6%). The etiology was uncertain in 62 (24%) of the ARDS patients. The microbiological work-up for these 62 patients is provided in Additional file 1: Table S3. Pneumonia and extra-pulmonary sepsis accounted for 60% of total cases and 79% of cases with identifiable etiology. For patients with non-ARDS hypoxemia, hydrostatic lung edema was the most common cause of hypoxemia (41.2%), followed by pneumonia (27.2%) and cancer (10.9%).

**Table 2:** Etiology of hypoxemic respiratory failure in cases with a PaO_2_/FiO_2_ ratio ≤ 300 mm Hg

Causes of hypoxemia		Etiologies of hypoxemia	n (%)
ARDS (n = 258)	Pulmonary ARDS, n = 137 (53.1%)	Pneumonia, total	125 (48.4)
		Bacterial pneumonia	87 (33.7)
		Viral pneumonia	16 (6.2)
		Fungal pneumonia	22 (8.5)
		Aspiration	8 (3.1)
		Vasculitis	4 (1.6)
	Extra-pulmonary ARDS, n = 59 (22.9%)	Extra-pulmonary sepsis	30 (11.6)
		Noncardiogenic shock	9 (3.5)
		Transfusion	6 (2.3)
		Drug toxicity	5 (1.9)
		Pancreatitis	3 (1.2)
		Burn	3 (1.2)
		Trauma	3 (1.2)
	Unclassified, n = 62 (24%)	Uncertain	62 (24)
Non-ARDS hypoxemia with PaO_2_/FiO_2_ ratios ≤ 300 (n = 294)		Hydrostatic lung edema	123 (41.8)
		Pneumonia	80 (27.2)
		Cancer, lung or metastatic cancer	32 (10.9)
		Pleural effusion or diseases	21 (7.1)
		Atelectasis	16 (5.4)
		Lung fibrosis	9 (3.1)
		Other	13 (4.4)

*ARDS* acute respiratory distress syndrome

Among pneumonia associated ARDS, bacterial, viral and fungal pneumonia accounted for 87 (70%), 16 (13%) and 22 (17%) cases, respectively. The bacterial pathogens are listed in Additional file 1: Table S4. Gram-negative bacteria accounted for 82.9% of bacterial infections and *Klebsiella* spp. were the most common pathogen. The 16 viral infection associated ARDS cases included 9 influenza and 7 cytomegalovirus pneumonia. The 22 fungal pneumonia associated ARDS cases included 15 *Pneumocystis jiroveci* and 7 aspergillosis pneumonia.

Cancer is the most common comorbidity in this ARDS cohort. Patients with cancer had higher SAPS II score (52 vs. 44, *p* < 0.001), higher levels of CRP (16 vs. 12 mg/dL, *p* = 0.006) and D-dimer (6.5 vs. 3.6 mg/L, *p* = 0.04), and lower platelet count (114 vs. 153 K/μL) as compared with patients without cancer (Additional file 1: Table S6). Table 3 shows the distribution of etiologies in patients with and without cancer to explore the potential influence of high proportion of cancer on etiology distribution. There was no significant difference between patients with and without cancer in the proportions of major etiologies, including pneumonia and extra-pulmonary sepsis. However, pancreatitis, burn and trauma associated ARDS was more commonly seen in patients without cancer. Additional File 1: Table S7 show the causes of severe hypoxemia in non-ARDS patients with and without cancer.

**Table 3:** Etiologies of ARDS in patients with and without cancer

Etiologies of ARDS	Cancer, n = 111	No cancer, n = 147	*p*-value
	n (%)	n (%)	
Pneumonia	55 (50)	70 (48)	0.76
Bacterial pneumonia	40 (73)	47 (67)	0.50
Influenza	4 (7)	5 (7)	1.0
*Pneumocystis jiroveci*	7 (13)	8 (11)	0.82
Other pathogens	4 (7)	10 (14)	0.22
Extra-pulmonary sepsis	16 (14)	14 (10)	0.23
Aspiration	3 (3)	5 (3)	1.0
Noncardiogenic shock	4 (4)	5 (3)	0.93
Transfusion	2 (2)	4 (3)	0.70
Drug toxicity	2 (2)	3 (2)	1.0
Other etiologies^a^	0 (0)	13 (9)	0.001
Uncertain	29 (26)	33 (22)	0.49

^a^Including pancreatitis, burn and trauma

### Etiology-associated heterogeneity

Table 4 shows the comparison between the major etiologies of ARDS in gas exchange abnormalities, respiratory mechanics, organ dysfunction, biomarkers of inflammation and coagulation, and outcome. Overall, the difference in respiratory parameters between major etiologies of ARDS was modest as compared with the differences in non-pulmonary organ dysfunction and outcome. For the two leading etiologies of ARDS, extra-pulmonary sepsis had more shock (48% versus 73%, *p* = 0.01), higher non-pulmonary SOFA scores (6 versus 9 points, *p* < 0.001), higher d-dimer levels (4.2 versus 9.7 mg/L, *p* = 0.02) and higher mortality (43% versus 67%, *p* = 0.02) than pneumonia associated ARDS. When bacterial pneumonia was compared with other non-bacterial pneumonia, there was significant difference in proportion of shock (*p* = 0.005) between bacterial and non-bacterial pneumonia.

**Table 4:** Comparison of gas exchange, organ dysfunction, biomarkers in coagulation and inflammation and mortality between the major etiologies of ARDS

Etiologies of ARDS	Pneumonia				Aspiration	Non-pulmonary sepsis	*p*-values^a^
	All pneumonia	Bacteria	Influenza	*Pneumocystis jiroveci*			
Respiratory parameters							
PaO_2_/FiO_2_ ratios	143 (99–194)	142 (94–197)	128 (73–144)	143 (116–266)	166 (120–183)	122 (87–194)	0.66
Ventilatory ratios	1.5 (1.2–2.0)	1.6 (1.1–2.1)	1.8 (1.7–2.2)	1.4 (1.2–1.8)	1.8 (1.4–2.3)	1.7 (1.3–2.2)	0.20
Static respiratory compliance, mL/cm H_2_O	32 (25–41)	30 (24–41)	34 (32–46)	33 (25–42)	30 (24–38)	29 (25–44)	0.92
Non-pulmonary organ dysfunction							
Vasopressor users, %	48 (39–57)	56 (45–67)	33 (7–70)	27 (8–55)	63 (24–91)	73 (54–88)	0.03
Non-pulmonary SOFA score	6 (3–8)	6 (3–9)	4 (2–7)	5 (2–8)	8 (5–11)	9 (6–12)	< 0.001
Biomarkers in inflammation and coagulation							
C-reactive protein, mg/dL	15.9 (8–23.1)	16.5 (9.7–25.7)	15.4 (6.6–22.1)	15.5 (8.5–18.8)	16 (1.9–19)	10.3 (2.8–16.5)	0.16
D-dimer, mg/L	4.2 (2.1–7.8)	4.9 (2.4–8.9)	5.7 (3.3–9.5)	1.9 (1.7–6.2)	17.3 (1–31.3)	9.7 (3.6–14.8)	0.07
Lactic acid, mmol/L	2.2 (1.5–3.5)	2.3 (1.5–4.2)	1.9 (1.5–2.1)	1.9 (1.1–2.4)	3.1 (2.5–13.5)	5.6 (2.6–8.4)	< 0.001
Outcome							
30-day mortality, %	43 (34–52)	43 (32–54)	22 (3–60)	53 (27–79)	63 (24–91)	67 (47–83)	0.04

Data were presented as median with interquartile range or proportions with 95% confidence intervals

^a^Between-group comparison among pneumonia, aspiration and non-pulmonary sepsis

**Fig. 2:**
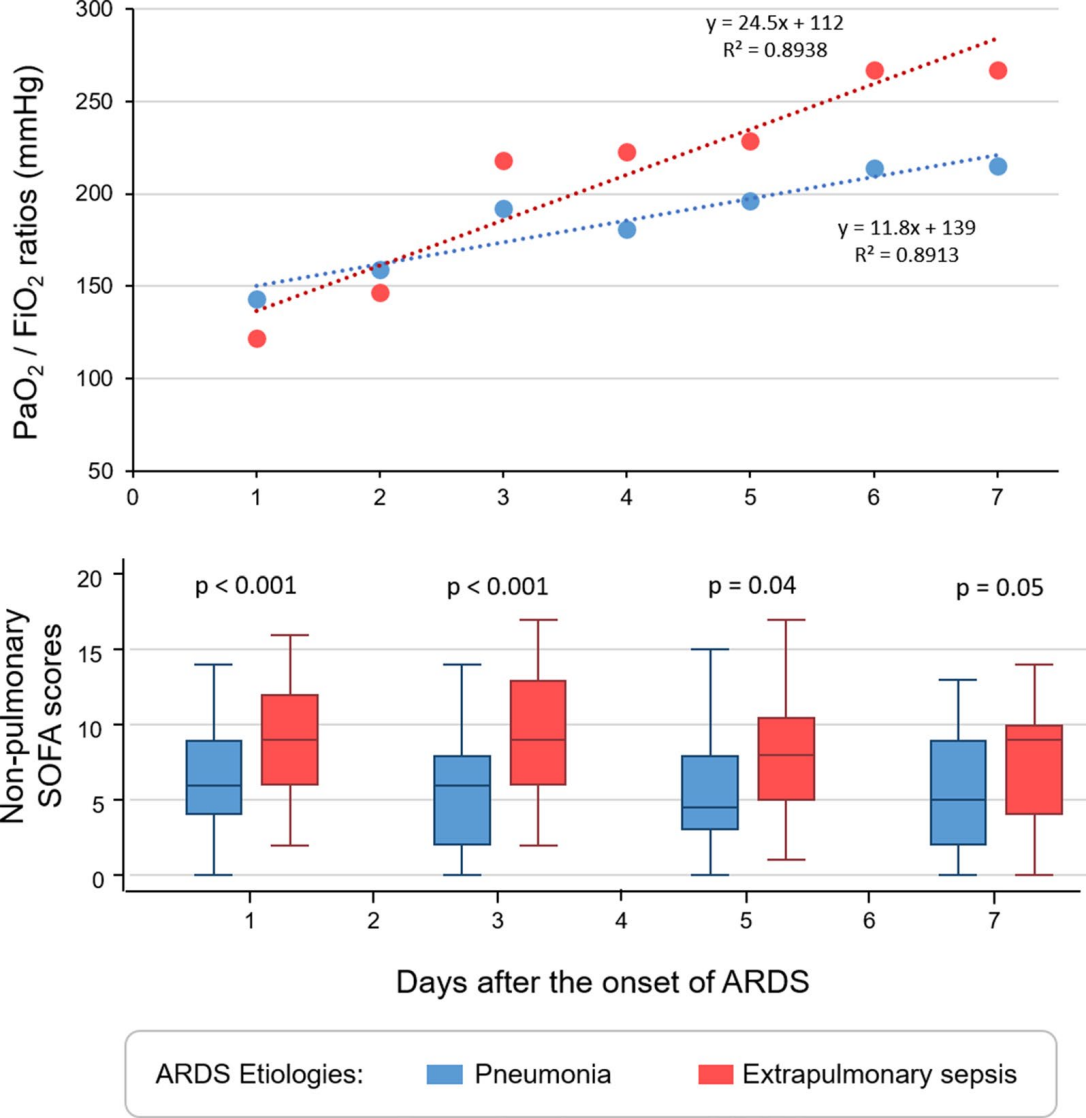
PaO_2_/FiO_2_ ratios and non-pulmonary organ dysfunction in the first seven days for the two primary etiologies of acute respiratory distress syndrome (ARDS)

Figure 2 shows the trajectories of the P/F ratios and non-pulmonary organ dysfunction during the first seven days for the two major etiologies of ARDS. Despite no difference on single-day observation of P/F ratios on day 1 (Table 4), the trajectory of P/F ratios differed between pneumonia and extra-pulmonary sepsis (Fig. 2). Extra-pulmonary sepsis associated ARDS demonstrated a significantly greater recovery rate in P/F ratios compared with pneumonia associated ARDS (difference = 13 mmHg/day, *p* = 0.01). In addition, extra-pulmonary sepsis associated ARDS had significantly higher non-pulmonary SOFA scores compared with pneumonia associated ARDS, especially in the first three days.

## Discussion

This study explored etiology-associated heterogeneity in ARDS and found that the trajectories of P/F ratios, hemodynamic instability, extra-pulmonary organ dysfunction, and mortality varied across etiologies of ARDS. This finding suggests that the etiology of ARDS could be used to identify a more homogeneous subsets of ARDS for prognostic and predictive enrichment, which are the recommended strategies by the Food and Drug Administration for increasing the efficiency of clinical trials across all fields [23]. Regarding prognostic enrichment, in line with a previous study [18], our study observed different mortality rates among ARDS patients due to a variety of etiologies (Table 4). This implicates that future studies of ARDS should report the outcomes not only for the entire cohort but also for each of the major etiology sub-groups to facilitate prognostic enrichment. In addition, between-study comparisons of outcomes should consider the effect of case-mix in etiologies [24]. Predictive enrichment refers to selecting patients more likely to respond to a given therapy. The differences in shock and organ dysfunction among major etiologies of ARDS in the present study suggests a potential effect modification by etiology in ARDS treatment. Previous data also showed that hemodynamic instability had a remarkable impact on the efficacy and safety of the open lung strategy in ARDS [19, 25]. In this regard, patients with ARDS due to etiologies more likely with hemodynamic instability may not benefit from a high PEEP strategy. Further studies are warranted to determine whether etiology of ARDS is an important effect modifier for ventilatory and pharmacological management of ARDS.

Pneumonia is the most common etiology of ARDS, and accounted for more than half of ARDS cases in previous cohort studies and clinical trials [4, 19]. Our data demonstrated that there was considerable within-group heterogeneity in pneumonia associated ARDS. Although bacteria are the major pathogen causing pneumonia, non-bacterial pathogens also play an important role in patients with comorbidities [26, 27]. Owing to population aging and the increasing usage of immunosuppressants, non-bacterial pneumonia in the ICU has become an emerging issue [26]. Previous studies and treatment guidelines for ARDS usually treat pneumonia as a single etiology of ARDS without distinguishing between non-bacterial pneumonia and bacterial pneumonia. Our data highlight the importance of differentiating between bacterial and non-bacterial pneumonia associated ARDS. Prospective large-scale studies are required to compare ARDS caused by bacterial pneumonia and major non-bacterial pathogens, such as influenza, cytomegalovirus and *Pneumocystis jiroveci*.

Phenotyping has been considered an important strategy for improving treatment outcomes in ARDS [16, 28]. Identifying the source of heterogeneity in ARDS is a crucial step in ARDS phenotyping. Several approaches have been proposed for ARDS phenotyping [8, 23]; a two-phenotype model based on plasma biomarkers identified two distinct subphenotypes of ARDS, which has clinical implications for prognostic and predictive enrichment [13]. The hyperinflammatory subphenotype has higher mortality and a different treatment response to PEEP and fluid management compared with the hypoinflammatory subphenotype [13, 21]. Other approaches include physiological factors and radiographic lung morphology based subgrouping [8, 14]. Etiology of ARDS is one of the clinical factors commonly used for ARDS subgrouping [15, 29]. However, the majority of studies adopt a dichotomous classification for subgrouping, such as pulmonary versus extrapulmonary ARDS or trauma versus non-trauma ARDS [17, 30]. Our data suggest that dichotomous classifications may not fully disclose the differences between major etiologies of ARDS. Dichotomous classification by pulmonary and extrapulmonary ARDS might just reflect the features of pneumonia and extrapulmonary sepsis because these two etiologies dominate pulmonary and extrapulmonary ARDS, respectively. Etiology-based management may help to improve the treatment outcomes of ARDS given the observed heterogeneity within etiology. In addition, subgrouping by etiology requires no additional blood tests or imaging examinations compared with other phenotyping methods.

Our study cohort had a higher proportion of cancer as compared with previous data [4, 14]. The high proportion of cancer could be attributable to the study settings since this study was conducted in a tertiary-care referral center which was responsible for accommodating many cancer patients. In fact, a similar comorbidity profile was also observed in the ARDS cohort from medical centers [31]. Cancer exerts a variety of direct and indirect impacts on risks and outcomes of ARDS [31, 32]. For instance, cancer patients with ARDS had a higher mortality rate compared to ARDS patients without cancer [31]. Cancer patients also more likely suffer from ARDS due to certain etiologies, such as bacterial, fungal and opportunistic infections [33]. The interpretation of our data with regard to the point estimates of etiology distribution and outcome should consider the influence from high proportion of cancer.

Our study did have several limitations. First, it was a single-center study, and the distribution of etiologies and outcome data may not be generalized to other institutions. Second, this study was underpowered to detect small between-group differences due to small sample size. Finally, we did not use multiplex polymerase chain reaction panels for the diagnosis of pneumonia and there was no universal protocol for the ARDS work-up during the study period. In addition, pneumonia pathogens such as fungus or PJP were not universally pursued. These shortcomings might have led to an underestimation of the prevalence of viral and other atypical pneumonia and an increase in the cases classified as uncertain etiology. Thus, our data should be interpreted with bearing this caveat in mind.

## Conclusion

Our study findings suggest that there was remarkable etiology-associated heterogeneity in ARDS. Heterogeneity was also observed within pneumonia associated ARDS when bacterial pneumonia was compared with other non-bacterial pneumonia. To develop tailored prognostic information and treatments for ARDS, future studies of ARDS should consider reporting etiology-specific data and exploring possible effect modification by etiology.

## Supplementary Information

**Additional file 1: Table S1**. Microbiological tests and biomarkers recorded in the study, and the proportion of ARDS patients receiving these tests. **Table S2**. Distribution of missing data for PaO2/FiO2 ratios and SOFA (Sequential Organ Failure Assessment) scores at different time points. **Table S3**. Microbiological tests for 62 cases of acute respiratory distress syndrome with unknown etiologies. **Table S4**. Species distribution of bacteria in 87 cases of bacterial pneumonia associated ARDS. **Table S5**. Etiologies of ARDS in patients with lung cancer and other cancer types. **Table S6**. Characteristics of patients with and without cancer in 258 patients with ARDS. **Table S7**. Causes of hypoxemia stratified by cancer in 294 non-ARDS patients with PaO2/FiO2 ratios ≤ 300 mmHg.

## Data Availability

The datasets used and/or analysed during the current study are available from the corresponding author on reasonable request.

## Abbreviations

ARDS: Acute respiratory distress syndrome
IQR: Inter-quartile range
PEEP: Positive end-expiratory pressure
P/F: PaO_2_/FiO_2_
SD: Standard deviation
SOFA: Sequential Organ Failure Assessment
