# Supplementary material for: Etiology-associated heterogeneity in acute respiratory distress syndrome: a retrospective cohort study
Source: BMC Pulm Med. 2021 May 31;21:183. doi: 10.1186/s12890-021-01557-9 (PMC8168042; doi:10.1186/s12890-021-01557-9)
Supplement: Supplementary file 1 — Additional file 1: Table S1. Microbiological tests and biomarkers recorded in the study, and the proportion of ARDS patients receiving these tests. Table S2. Distribution of missing data for PaO2/FiO2 ratios and SOFA (Sequential Organ Failure Assessment) scores at different time points. Table S3. Microbiological tests for 62 cases of acute respiratory distress syndrome with unknown etiologies. Table S4. Species distribution of bacteria in 87 cases of bacterial pneumonia associated ARDS. Table S5. Etiologies of ARDS in patients with lung cancer and other cancer types. Table S6. Characteristics of patients with and without cancer in 258 patients with ARDS. Table S7. Causes of hypoxemia stratified by cancer in 294 non-ARDS patients with PaO2/FiO2 ratios ≤ 300 mmHg. [file 12890_2021_1557_MOESM1_ESM.pdf]

Additional file 1

Etiology-associated heterogeneity in acute respiratory distress syndrome: a retrospective cohort study

Sheng-Yuan Ruan, Chun-Ta Huang, Ying-Chun Chien, Chun-Kai Huang, Jung-Yien Chien, Lu-Cheng Kuo, Ping-Hung Kuo, Shih-Chi Ku and Huey-Dong Wu

	Contents	Page
**Table S1.**	Microbiological tests and biomarkers recorded in the study, and the proportion of ARDS patients receiving these tests ………………………………………………………………………………	2
**Table S2.**	Distribution of missing data for PaO2/FiO2 ratios and SOFA (Sequential Organ Failure Assessment) scores at different time points …………………………………………………………………..	3
**Table S3.**	Microbiological tests for 62 cases of acute respiratory distress syndrome with unknown etiologies ……………………	4
**Table S4.**	Species distribution of bacteria in 87 cases of bacterial pneumonia associated ARDS……………………………………….	5
**Table S5.**	Etiologies of ARDS in patients with lung cancer and other cancer types……………………………………………………………….	6
**Table S6.**	Characteristics of patients with and without cancer in 258 patients with ARDS…………………………………………………….	7
**Table S7.**	Causes of hypoxemia stratified by cancer in 294 non-ARDS patients with PaO_2/FiO_2 ratios ≤ 300 mmHg……………………	8

Table S1. Microbiological tests and biomarkers recorded in the study, and the proportion of ARDS patients receiving these tests (n=258).

Laboratory tests	Tested patients, n	%
**Sputum, or nasopharyngeal aspirate/swab**		
Sputum smear & Gram stain	249	96.5
Sputum bacterial culture	248	96.1
Acid fast stain & Mycobacterium culture	212	82.2
Influenza rapid test or PCR	92	35.7
Influenza A+B rapid screening test	67	26.0
Influenza virus A & B real-time PCR	56	21.7
Chlamydiae antigen	132	51.2
Pnemocystis jirovecii real-time PCR	57	22.1
**Microbiology, serum or plasma**		
Mycoplasma pneumoniae IgM Ab	141	54.7
Plasma cytomegalovirus real-time PCR	79	30.6
Aspergillus antigen detection (ELISA)	106	41.1
**Microbiology, urine**		
Legionella antigen test	143	55.4
**Serum or plasma (Day 1)**		
C-reactive protein	178	69.0
D-dimer	107	41.5
Lactic acid	218	84.5
Albumin	192	74.4

ELISA, enzyme-linked immunosorbent assay; PCR, polymerase chain reaction

Table S2. Distribution of missing data for PaO_2/FiO_2 ratios and SOFA (Sequential Organ Failure Assessment) scores at different time points.

Variables	ARDS		Non-ARDS	
	No.	Missing data	No.	Missing data
**PaO_2/FiO_2 ratio**				
Day 1	258	0 (0%)	294	0 (0%)
Day 2	254	11 (4.3%)	292	37 (12.7%)
Day 3	245	20 (8.2%)	286	53 (18.5%)
Day 4	243	22 (9.1%)	277	68 (24.5%)
Day 5	239	33 (13.8%)	264	63 (23.9%)
Day 6	236	40 (16.9%)	263	85 (32.3%)
Day 7	228	52 (22.8%)	255	89 (34.9%)
**SOFA, central nervous system**				
Day 1	258	9 (3.5%)	294	17 (5.8%)
Day 3	245	10 (4.1%)	286	21 (7.3%)
Day 5	239	10 (4.2%)	264	22 (8.3%)
Day 7	228	14 (6.1%)	255	30 (11.8%)
**SOFA, cardiovascular system**				
Day 1	258	0 (0%)	294	0 (0%)
Day 3	245	0 (0%)	286	0 (0%)
Day 5	239	0 (0%)	264	0 (0%)
Day 7	228	0 (0%)	255	0 (0%)
**SOFA, liver function**				
Day 1	258	48 (18.6%)	294	57 (19.4%)
Day 3	245	94 (38.4%)	286	129 (45.1%)
Day 5	239	111 (46.4%)	264	137 (51.9%)
Day 7	228	97 (42.5%)	255	132 (51.8%)
**SOFA, coagulation**				
Day 1	258	5 (1.9%)	294	12 (4.1%)
Day 3	245	3 (1.2%)	286	13 (4.5%)
Day 5	239	6 (2.5%)	264	20 (7.6%)
Day 7	228	13 (5.7%)	255	31 (12.2%)
**SOFA, renal function**				
Day 1	258	9 (3.5%)	294	20 (6.8%)
Day 3	245	24 (9.8%)	286	34 (11.9%)
Day 5	239	39 (16.3%)	264	49 (18.6%)
Day 7	228	33 (14.5%)	255	58 (22.7%)

Table S3. Microbiological tests for 62 cases of acute respiratory distress syndrome with unknown etiologies.

Specimen	Tested patients, n	%
**Sputum, or nasopharyngeal aspirate/swab**		
Sputum smear & Gram stain	60	96.8
Sputum bacterial culture	60	96.8
Acid fast stain & Mycobacterium culture	52	83.9
Influenza rapid test or real-time PCR	33	53.2
Chlamydiae antigen	45	72.6
Pnemocystis jirovecii real-time PCR	19	30.6
**Serum or plasma**		
Mycoplasma pneumoniae IgM Ab	45	72.6
Plasma cytomegalovirus real-time PCR	18	29.0
Aspergillus antigen detection (ELISA)	30	48.4
**Urine**		
Legionella antigen test	45	72.6
Pneumococcal antigen test	11	17.7

ELISA, enzyme-linked immunosorbent assay; PCR, polymerase chain reaction

Table S4. Species distribution of bacteria in 87 cases of bacterial pneumonia associated ARDS. Sputum cultures yielded two bacterial species in 27 cases and culture data were unavailable in 3 cases.

Bacteria species	Case number	%
**Gram-negative bacteria**	92	82.9
Klebsiella spp.	23	20.7
Acinetobacter spp.	15	13.5
Stenotrophomonas maltophilia	12	10.8
Pseudomonas aeruginosa	11	9.9
Escherichia coli	7	6.3
Enterobacter spp.	5	4.5
Burkholderia spp.	5	4.5
Legionella spp.	5	4.5
Others	9	8.1
**Gram-positive bacteria**	19	17.1
Staphylococcus aureus	13	11.7
Others	6	5.4

Table S5. Etiologies of ARDS in patients with lung cancer and other cancer types.

**Patient groups**	**Major ARDS Etiologies (%)**		**Mortality**
Lung cancer (n = 26)	Pneumonia	(42%)	50%
	Extra-pulmonary sepsis	(8%)	
	Aspiration	(4%)	
	Noncardiogenic shock	(8%)	
Other cancer (n = 85)	Pneumonia	(52%)	45%
	Extra-pulmonary sepsis	(16%)	
	Aspiration	(2%)	
	Noncardiogenic shock	(2%)	

Table S6. Characteristics of patients with and without cancer in 258 patients with ARDS.

Variables	Cancer n = 111	No cancer n = 147	p-value
Age, yr, median (IQR)	64 (55-72)	68 (55-79)	0.15
Sex, female, n (%)	35 (32)	48 (33)	0.85
SAPS II score, median (IQR)	52 (43-61)	44 (37-52)	<0.001
Respiratory parameters, median (IQR)			
FiO_2	0.6 (0.5-1.0)	0.7 (0.5-1.0)	0.74
PaO_2/FiO_2 ratio, mm Hg	149 (99-210)	142 (98-195)	0.39
PEEP, cm H_2O	8 (6-10)	8 (6-10)	0.50
pH	7.43 (7.38-7.46)	7.41 (7.36-7.45)	0.02
$PaCO_2$, mm Hg	32 (27-37)	32 (28-38)	0.45
HCO_3^-, mmol/L	21 (18-25)	20 (18-24)	0.85
Tidal volume/pBW, mL/kg	8.5 (7.2-10.7)	8.7 (7.3-10.0)	0.69
Minute ventilation, L/min	10.3 (8.3-13.7)	10.5 (8.1-12.9)	0.52
Respiratory compliance, mL/cm H_2O	30 (25-45)	32 (24-40)	0.45
Respiratory resistance, cm $H_2O{\cdot}s/L$	14 (11-17)	15(12-18)	0.40
Biomarkers, median (IQR)			
C-reactive protein, mg/dL	16 (10-25)	12 (7-19)	0.006
Platelet, K/μL	114 (66-183)	153 (86-218)	0.03
D-dimer, mg/L	6.5 (2.8-12.5)	3.6 (2.1-8.9)	0.04
Lactic acid, mmol/L	2.2 (1.5-3.8)	2.3 (1.5-4.6)	0.67
Albumin, g/dL	2.7 (2.3-2.9)	2.7 (2.3-3.0)	0.37
30-day mortality, n (%)	51 (50)	61 (42)	0.48

Table S7. Causes of hypoxemia stratified by cancer in 294 non-ARDS patients with PaO_2/FiO_2 ratios ≤ 300 mmHg.

**Causes of hypoxemia**	**Comorbid cancer**	
	**Yes**	**No**
	**n = 106**	**n = 188**
Hydrostatic lung edema	19 (18%)	104 (55%)
Pneumonia	28 (26%)	52 (28%)
Primary or metastatic lung cancer	32 (30%)	0 (0%)
Pleural effusion or diseases	14 (13%)	7 (4%)
Atelectasis	8 (8%)	8 (4%)
Other	5 (5%)	17 (9%)
